# Supplementary material for: Discriminatory attitude towards people living with HIV/AIDS and its associated factors among adult population in 15 sub-Saharan African nations
Source: PLoS One. 2022 Feb 4;17(2):e0261978. doi: 10.1371/journal.pone.0261978 (PMC8815885; doi:10.1371/journal.pone.0261978)
Supplement: S1 Table — (DOCX) [file pone.0261978.s001.docx]

**S1 Table**. Sociodemographic characteristics of respondents by regions of Africa

| **Variables** | **Regions of Africa accounted for this study** | | | | | |
| --- | --- | --- | --- | --- | --- | --- |
|  | **Eastern** | | **Western** | | **Central** | |
|  | **Frequency (N=145,797)** | **Percentage** | **Frequency (N=146,775)** | **Percentage** | **Frequency (N=35,971)** | **Percentage** |
| Sex  Male  Female | 49,029  96,768 | 33.63  66.37 | 38,342  98,076 | 28.11  71.89 | 10,951  25,020 | 30.44  69.56 |
| Age  15-19  20-24  25-29  30-34  35-39  40-44  45-49 | 32,811  27,642  24,050  20,880  17,165  13,317  9,932 | 22.50  18.96  16.50  14.32  11.77  9.13  6.81 | 28,179  22,787  23,413  19,303  18,132  13,137  11,467 | 29.66  16.70  17.16  14.15  13.29  9.63  8.41 | 8,760  6,978  6,274  4,739  3,823  3,049  2,348 | 24.35  19.40  17.44  13.17  10.63  8.48  6.53 |
| Educational status  No education  Primary  Secondary  Higher | 23,912  66,810  46,394  8,681 | 16.40  45.82  31.82  5.95 | 51,459  21,108  52,070  11,781 | 37.72  15.47  38.17  8.64 | 5,208  10,283  17,643  2,837 | 14.48  28.59  49.05  7.89 |
| Occupation  Not working  Working | 49,360  96,437 | 33.85  66.15 | 42,038  94,380 | 30.82  69.18 | 12,180  23,791 | 33.86  66.14 |
| Marital status  Single  Married  Widowed/separated/divorced | 46,840  85,733  13,224 | 32.13  58.80  9.07 | 43,416  86,956  6,046 | 31.83  63.74  4.43 | 14,552  18,722  2,697 | 40.45  52.05  7.50 |
| Wealth status  Poorest  Poorer  Middle  Richer  Richest | 24,877  26,481  27,746  30,496  36,197 | 17.06  18.16  19.03  20.92  24.83 | 22,260  24,653  26,026  29,970  33,509 | 16.32  18.07  19.08  21.97  24.56 | 4,873  5,737  7,319  8,443  9,599 | 13.55  15.95  20.35  23.47  26.69 |
| Sex of household head  Male  Female | 108,851  36,946 | 74.66  25.34 | 110,111  26,307 | 80.72  19.28 | 26,270  9,700 | 73.03  26.97 |
| Contraceptive use  No  Yes | 93,352.3107  52,444.613 | 64.03  35.97 | 111,449  24,969 | 81.70  18.30 | 27,874  8,096 | 77.49  22.51 |
| Media exposure  No  Yes | 47,667  98,130 | 32.69  67.31 | 36,083  100,335 | 26.45  73.55 | 9,125  26,845 | 25.37  74.63 |
| Comprehensive knowledge of HIV/AIDS  No  Yes | 76,949  68,848 | 52.78  47.22 | 88,338  48,080 | 64.76  35.24 | 21,210  14,760 | 58.97  41.03 |
| Residence  Urban  Rural | 38,910  106,887 | 26.69  73.31 | 67,354  69,064 | 49.37  50.63 | 23,860  12,110 | 66.33  33.67 |
| Community-level of women literacy  Low  High | 71,655  74,142 | 49.15  50.85 | 75,211  61,207 | 55.13  44.87 | 18,502  17,468 | 51.44  48.56 |
| Community-level media exposure  Low  High | 77,765  68,032 | 53.34  46.66 | 76,223  60,195 | 55.87  44.13 | 16,570  19,400 | 46.07  53.93 |
