# Supplementary material for: Discriminatory attitude towards people living with HIV/AIDS and its associated factors among adult population in 15 sub-Saharan African nations
Source: PLoS One. 2022 Feb 4;17(2):e0261978. doi: 10.1371/journal.pone.0261978 (PMC8815885; doi:10.1371/journal.pone.0261978)
Supplement: S2 Table — (DOCX) [file pone.0261978.s002.docx]

**S2 Table.** Multilevel analysis for assessing factors associated with discriminatory attitude towards people living with HIV/AIDS among reproductive age women and men, analyzed separately.

1. **Multilevel analysis of factors associated with discriminatory attitude towards people living with HIV/AIDS among reproductive age women**

| Variables | Model 1 | Model 2 | Model 3 | Model 4 |
| --- | --- | --- | --- | --- |
| Age  15-19  20-24  25-29  30-34  35-39  40-44  45-49 |  | 0.79 (0.76, 0.83)  0.76 (0.72, 0.79)  0.65 (0.62, 0.68)  0.66 (0.63, 0.69)  0.60 (0.56, 0.63)  0.63 (0.60, 0 .67) |  | 1.00  0.79 (0.76, 0.82)***  0.78(0.72, 0.79)***  0.65 (0.62, 0.68)***  0.65 (0.62, 0.69)***  0.60 (0.56 0.63)***  0.64 (0.60, 0.68)*** |
| Educational status  No education  Primary  Secondary  Higher |  | 1.00  0.39 (0.37, 0.41)  0.36 (0.34, 0.38)  0.26 (0.24, 0.28) |  | 1.00  0.42 (0.40, 0.44)***  0.37 (0.35, 0.39)***  0.26 (0.24, 0.28)*** |
| Occupation  Not working  Working |  | 1.00  1.00 (0.96, 1.03) |  | 1.00  1.01 (0.97, 1.04) |
| Marital status  Single  Married  Widowed/separated/divorced |  | 1.00  1.00 (0.96, 1.04)  0.70 (0.66, 0.74) |  | 1.00  0.99 (.951304 1.029)  0.70 (.6601215 .74)*** |
| Wealth status  Poorest  Poorer  Middle  Richer  Richest |  | 1.00  0.93 (0.89, 0.98)  0.85 (0.81, 0.90)  0.77 (0.72, 0.82)  0.62 (0.57, 0.66) |  | 1.00  0.91(0.87, 0.96)***  0.80 (0.75, 0.85)***  0.65 (0.61, 0.70)***  0.47 (0.44, 0.51)*** |
| Sex of household head  Male  Female |  | 1.00  0.89 (0.86, 0.92) |  | 1.00  0.89 (0.86, 0.92)*** |
| Contraceptive use  No  Yes |  | 1.00  0.73 (0.71, 0.76) |  | 1.00  0.74 (0.72, 0.77)*** |
| Media exposure  No  Yes |  | 1.00  1.05 (1.01, 1.09) |  | 1.00  0.99 (0.95, 1.03) |
| Comprehensive knowledge of HIV/AIDS  No  Yes |  | 1.00  0.38 (0.37, 0.39) |  | 1.00  0.39 (0.37, 0.40)*** |
| Residence  Urban  Rural |  |  | 1.00  1.27 (1.18, 1.36) | 1.00  0.65 (0.60, 0.69)*** |
| Country  West SSA  Central SSA  East SSA |  |  | 1.00  0.48 (0.44, 0.52)  0.54 (0.51, 0.58) | 1.00  0.51 (0.47, 0.55)***  0.73 (0.68, 0.77)*** |
| Community-level of women literacy  Low  High |  |  | 1.00  1.03 (0.86, 1.22) | 1.00  0.84 (0.71, 0.99)* |
| Community-level media exposure  Low  High |  |  | 1.00  0.99 (0.83, 1.19) | 1.00   - 1. 0.89, 1.25) |

Note: ***=P value<0.001, *=P value<0.05

1. **Multilevel analysis of factors associated with discriminatory attitude towards people living with HIV/AIDS among reproductive age men.**

| Variables | Model 1 | Model 2 | Model 3 | Model 4 |
| --- | --- | --- | --- | --- |
| Age  15-19  20-24  25-29  30-34  35-39  40-44  45-49 |  | 1.00  0.82 (0.77, 0.87)  0.80 (0.74, 0.86)  0.76 (0.70, 0.83)  0.77 (0.71, 0.84)  0.76 (0.70, 0.84)  0.77 (0.70, 0.84) |  | 1.00  0.80 (0.75, 0.85)***  0.76 (0.71, 0.82)***  0.72 (0.66, 0.78)***  0.71 (0.65, 0.77)***  0.71 (0.65, 0.79)** |
| Educational status  No education  Primary  Secondary  Higher |  | 1.00  0.42 (0.40, 0.45)  0.39 (0.36, 0.41)  0.31 (0.28, 0.34) |  | 1.00  0.49 (0.45, 0.52)***  0.39 (0.37, 0.42)***  0.30 (0.27, 0.33)*** |
| Occupation  Not working  Working |  | 1.00  1.12 (1.06, 1.19) |  | 1.00  1.10 (1.04, 1.16)** |
| Marital status  Single  Married  Widowed/separated/divorced |  | 1.00  0.78 (0.73, 0.83)  0.77 (0.69, 0.87) |  | 1.00  0.82 (0.77, 0.87)***  0.83 (0.74, 0.94)** |
| Wealth status  Poorest  Poorer  Middle  Richer  Richest |  | 1.00  0.89 (0.83, 0.95)  0.77 (0.72, 0.83)  0.72 (0.66, 0.78)  0.64 (0.59, 0.71) |  | 1.00  0.89 (0.81, 0.94)***  0.73 (0.68, 0.79)***  0.63 (0.58, 0.69)***  0.52 (0.47, 0.58)*** |
| Sex of household head  Male  Female |  | 1.00  0.92 (0.87, 0.97) |  | 1.00  0.95 (0.89, 1.01) |
| Contraceptive use  No  Yes |  | 1.00  0.67 (0.64, 0.70) |  | 1.00  0.72 (0.68, 0.75)*** |
| Media exposure  No  Yes |  | 1.00  0.91 (0.86, 0.97) |  | 1.00  0.83 (0.78, 0.88)*** |
| Comprehensive knowledge of HIV/AIDS  No  Yes |  | 1.00  0.38 (0.36, 0.39) |  | 1.00  0.40 (0.38, 0.42)*** |
| Residence  Urban  Rural |  |  | 1.00  1.34 (1.24, 1.44) | 1.00  0.73 (0.67, 0.79)*** |
| Country  West SSA  Central SSA  East SSA |  |  | 1.00  0.54 (0.49, 0.60)  0.39 (0.36, 0.42) | 1.00  0.56 (0.51, 0.62)***  0.48 (0.45, 0.52)*** |
| Community-level of women literacy  Low  High |  |  | 1.00  1.05 (0.94, 1.17) | 1.00  0.84 (0.75, 0.93)*** |
| Community-level media exposure  Low  High |  |  | 1.00  1.06 (0.95, 1.19) | 1.00  1.20 (1.08, 1.34)*** |

Note: ***=P value<0.001, **=P value<0.01
