# Supplementary material for: Discriminatory attitude towards people living with HIV/AIDS and its associated factors among adult population in 15 sub-Saharan African nations
Source: PLoS One. 2022 Feb 4;17(2):e0261978. doi: 10.1371/journal.pone.0261978 (PMC8815885; doi:10.1371/journal.pone.0261978)
Supplement: S3 Table — (DOCX) [file pone.0261978.s003.docx]

**S3 Table. Multilevel analysis for assessing factors associated with discriminatory attitude towards people living with HIV/AIDS by regions of sub-Saharan Africa**

1. **Multilevel analysis for assessing factors associated with discriminatory attitude towards people living with HIV/AIDS in Western Africa.**

| Variables | Model 1 | Model 2 | Model 3 | Model 4 |
| --- | --- | --- | --- | --- |
| Sex  Male  Female |  | 1.00  1.01 (0.96, 1.06) |  | 1.00  1.00 (0.95, 1.05) |
| Age  15-19  20-24  25-29  30-34  35-39  40-44  45-49 |  | 1.00  0.83 (0.79, 0.87)  0.84 (0.79, 0.89)  0.75 (0.70, 0.79)  0.74 (0.69, 0.79)  0.69 (0.64, 0.74)  0.70 (0.65, 0.75) |  | 1.00  0.81 (0.77, 0.85)***  0.80 (0.75, 0.84)***  0.71 (0.66, 0.75)***  0.69 (0.65, 0.74) ***  0.64 (0.60, 0.69)***  0.65 (0.61, 0.71)*** |
| Educational status  No education  Primary  Secondary  Higher |  | 1.00  0.37 (0.35, 0.39)  0.47 (0.45, 0.50)  0.33 (0.30, 0.36) |  | 1.00  0.38 (0.36, 0.40)***  0.45 (0.42, 0.47)***  0.30 (0.28, 0.33)*** |
| Occupation  Not working  Working |  | 1.00  1.11 (1.06, 1.16) |  | 1.00  1. 12 (1.07, 1.17)*** |
| Marital status  Single  Married  Widowed/separated/divorced |  | 1.00  0.84 (0.79, 0.89)  0.60 (0.56, 0.65) |  | 1.00  0.88 (0.84, 0.93)***  0.62 (0.57, 0.67)*** |
| Wealth status  Poorest  Poorer  Middle  Richer  Richest |  | 1.00  0.86 (0.81, 0.91)  0.76 (0.71, 0.82)  0.69 (0.63, 0.74)  0.58 (0.53, 0.63) |  | 1.00  0.83 (0.78, 0.88)***  0.68 (0.64, 0.73)***  0.54 (0.50, 0.59)***  0.40 (0.36, 0.44)*** |
| Sex of household head  Male  Female |  | 1.00  0.97 (0.92, 1.01) |  | 1.00  0.95 (0.91, 0.99)* |
| Contraceptive use  No  Yes |  | 1.00  0.61 (0.58, 0.64) |  | 1.00  0.63 (0.60, 0.66)*** |
| Media exposure  No  Yes |  | 1.00  1.18 (1.12, 1.24) |  | 1.00  1.12 (1.07, 1.18)*** |
| Comprehensive knowledge of HIV/AIDS  No  Yes |  | 1.00  0.42 (0.40, 0.44) |  | 1.00  0.41 (0.40, 0.44)*** |
| Residence  Urban  Rural |  |  | 1.00  0.83 (0.75, 0.92) | 1.00  0.49 (0.45, 0.54)*** |
| Community-level of women literacy  Low  High |  |  | 1.00  1.33 (1.13, 1.55) | 1.00  1.05 (0.92, 1.21) |
| Community-level media exposure  Low  High |  |  | 1.00  1.05 (0.89, 1.23) | 1.00  1.10 (0.96, 1.25) |

Note: ***=P value<0.001, *=P value<0.05

1. **Multilevel analysis for assessing factors associated with discriminatory attitude towards people living with HIV/AIDS in Eastern Africa**

| Variables | Model 1 | Model 2 | Model 3 | Model 4 |
| --- | --- | --- | --- | --- |
| Sex  Male  Female |  | 1.00  1.14 (1.08, 1.19) |  | 1.00  1.14 (1.08, 1.19)*** |
| Age  15-19  20-24  25-29  30-34  35-39  40-44  45-49 |  | 1.00  0.78 (0.74, 0.82)  0.67 (0.63, 0.72)  0.58 (0.54, 0.63)  0.58 (0.55, 0.63)  0.56 (0.52, 0.61)  0.61 (0.56, 0.66) |  | 1.00  0.77 (0.73, 0.82)***  0.66 (0.62, 0.71)***  0.58 (0.54, 0.62)  0.58 (0.54, 0.62)***  0.57 (0.51, 0.60)***  0.60 (0.56, 0.65)*** |
| Educational status  No education  Primary  Secondary  Higher |  | 1.00  0.46 (0.43, 0.48)  0.32 (0.30, 0.34)  0.22 (0.20, 0.25) |  | 1.00  0.46 (0.43, 0.48)***  0.31 (0.29, 0.33)***  0.21 (0.19, 0.24)*** |
| Occupation  Not working  Working |  | 1.00  0.92 (0.87, 0.96) |  | 1.00  0.92 (0.88, 0.97)** |
| Marital status  Single  Married  Widowed/separated/divorced |  | 1.00  101 (0.96, 1.06)  0.79 (0.73, 0.85) |  | 1.00  1.01 (0.96, 1.07)  0.78 (0.73, 0.85)*** |
| Wealth status  Poorest  Poorer  Middle  Richer  Richest |  | 1.00  0.98 (0.92, 1.04)  0.92 (0.85, 0.99)  0.88 (0.81, 0.97)  0.77 (0.69, 0.85) |  | 1.00  0.98 (0.92, 1.04)  0.91 (0.84, 0.98)*  0.81 (0.74, 0.89)***  0.65 (0.58, 0.73)*** |
| Sex of household head  Male  Female |  | 1.00  0.89 (0.86, 0.94) |  | 1.00  0.88 (0.85, 0.93)*** |
| Contraceptive use  No  Yes |  | 1.00  0.92 (0.88, 0.95) |  | 1.00  0.91 (0.88, 0.95)*** |
| Media exposure  No  Yes |  | 1.00  0.79 (0.75, 0.83) |  | 1.00  0.79 (0.75, 0.82)*** |
| Comprehensive knowledge of HIV/AIDS  No  Yes |  | 1.00  0.38 (0.36, 0.39) |  | 1.00  0.38 (0.36, 0.40)*** |
| Residence  Urban  Rural |  |  | 1.00  1.55 (1.38, 1.75) | 1.00  0.78 (0.69, 0.87)*** |
| Community-level of women literacy  Low  High |  |  | 1.00  1.06 (0.99, 1.15) | 1.00  1.01(0.94, 1.08) |
| Community-level media exposure  Low  High |  |  | 1.00  0.86 (0.80, 0.93) | 1.00   - 1. 0.85, 0.98)* |

Note: ***=P value<0.001, **=P value<0.01, *=P value<0.05

1. **Multilevel analysis for assessing factors associated with discriminatory attitude towards people living with HIV/AIDS in Central Africa.**

| Variables | Model 1 | Model 2 | Model 3 | Model 4 |
| --- | --- | --- | --- | --- |
| Sex  Male  Female |  | 1.00  0.76 (0.69, 0.84) |  | 1.00  0.76 (0.69, 0.84)*** |
| Age  15-19  20-24  25-29  30-34  35-39  40-44  45-49 |  | 1.00  0.75 (0.68, 0.82)  0.68 (0.61, 0.75)  0.57 (0.50, 0.64)  0.59 (0.52, 0.67)  0.55 (0.48, 0.63)  0.55 (0.47, 0.64) |  | 1.00  0.75 (0.69, 0.82)***  0.68 (0.62, 0.76)***  0.57 (0.50, 0.65)***  0.59 (0.52, 0.66)***  0.55 (0.48, 0.63)***  0.55 (0.47, 0.64)*** |
| Educational status  No education  Primary  Secondary  Higher |  | 1.00  0.77 (0.70, 0.86)  0.45 (0.41, 0.50)  0.35 (0.29, 0.42) |  | 1.00  0.77 (0.70, 0.86)***  0.45 (0.41, 0.51)***  0.35 (0.29, 0.42)*** |
| Occupation  Not working  Working |  | 1.00  0.99 (0.92, 1.08) |  | 1.00  0.99 (0.91, 1.08) |
| Marital status  Single  Married  Widowed/separated/divorced |  | 1.00  1.03 (0.93, 1.13)  0.92 (0.81, 1.05) |  | 1.00  1.03 (0.93, 1.13)  0.93 (0.81, 1.06) |
| Wealth status  Poorest  Poorer  Middle  Richer  Richest |  | 1.00  0.82 (0.72, 0.94)  0.64 (0.55, 0.74)  0.51 (0.43, 0.61)  0.46 (0.38, 0.55) |  | 1.00  0.85 (0.75, 0.97)*  0.69 (0.59, 0.81)***  0.57 (0.47, 0.68)***  0.51 (0.42, 0.63)*** |
| Sex of household head  Male  Female |  | 1.00  0.90 (0.83, 0.98) |  | 1.00  0.91 (0.84, 0.99)* |
| Contraceptive use  No  Yes |  | 1.00  0.85 (0.78, 0.93) |  | 1.00  0.85 (0.78, 0.93)*** |
| Media exposure  No  Yes |  | 1.00  0.97 (0.88, 1.06) |  | 1.00  0.98 (0.89, 1.07) |
| Comprehensive knowledge of HIV/AIDS  No  Yes |  | 1.00  0.44 (0.40, 0.48) |  | 1.00  0.44 (0.40, 0.47)*** |
| Residence  Urban  Rural |  |  | 1.00  2.31 (1.95, 2.73) | 1.00  1.15 (0.99, 1.33) |
| Community-level of women literacy  Low  High |  |  | 1.00  1.07 (0.95, 1.21) | 1.00  0.99 (0.89, 1.09) |
| Community-level media exposure  Low  High |  |  | 1.00  0.80 (0.71, 0.91) | 1.00  0.89 (0.80, 0.99)* |

Note: ***=P value<0.001, *=P value<0.05
